# TMEM16A/F support exocytosis but do not inhibit Notch-mediated goblet cell metaplasia of BCi-NS1.1 human airway epithelium

**DOI:** 10.3389/fphys.2023.1157704

**Published:** 2023-05-09

**Authors:** Raquel Centeio, Inês Cabrita, Rainer Schreiber, Karl Kunzelmann

**Affiliations:** ^1^ Physiological Institute, University of Regensburg, Regensburg, Germany; ^2^ Department II of Internal Medicine and Center for Molecular Medicine Cologne, Faculty of Medicine and University Hospital Cologne, University of Cologne, Cologne, Germany

**Keywords:** TMEM16F, exocytosis, goblet cell metaplasia, Notch signaling, TMEM16A

## Abstract

Cl^−^ channels such as the Ca^2+^ activated Cl^−^ channel TMEM16A and the Cl^−^ permeable phospholipid scramblase TMEM16F may affect the intracellular Cl^−^ concentration ([Cl^−^]_i_), which could act as an intracellular signal. Loss of airway expression of TMEM16A induced a massive expansion of the secretory cell population like goblet and club cells, causing differentiation into a secretory airway epithelium. Knockout of the Ca^2+^-activated Cl^−^ channel TMEM16A or the phospholipid scramblase TMEM16F leads to mucus accumulation in intestinal goblet cells and airway secretory cells. We show that both TMEM16A and TMEM16F support exocytosis and release of exocytic vesicles, respectively. Lack of TMEM16A/F expression therefore causes inhibition of mucus secretion and leads to goblet cell metaplasia. The human basal epithelial cell line BCi-NS1.1 forms a highly differentiated mucociliated airway epithelium when grown in PneumaCult™ media under an air liquid interface. The present data suggest that mucociliary differentiation requires activation of Notch signaling, but not the function of TMEM16A. Taken together, TMEM16A/F are important for exocytosis, mucus secretion and formation of extracellular vesicles (exosomes or ectosomes) but the present data do no not support a functional role of TMEM16A/F in Notch-mediated differentiation of BCi-NS1.1 cells towards a secretory epithelium.

## Introduction

Signaling by chloride (Cl^−^) ions affects numerous cellular functions (Valdivieso Á and Santa-Coloma, 2019; Lüscher et al., 2020). Cl^−^ channels such as the Ca^2+^ activated Cl^−^ channel TMEM16A and the Cl^−^ permeable phospholipid scramblase TMEM16F determine the intracellular Cl^−^ concentration, and may couple intracellular Cl^−^ concentration to membrane remodeling and cellular morphogenesis (He et al., 2017; Hong et al., 2019). Previous reports provided evidence for a role of TMEM16F in membrane shedding and release of membrane microvesicles (Sommer et al., 2016; Veit et al., 2018; Bleibaum et al., 2019). Bricogne et al. (2019) demonstrated membrane expansion by Ca^2+^ dependent activation of TMEM16F and extensive shedding of ectosomes, while Cabrita et al. (2019) demonstrated the role of TMEM16F for cellular exocytosis. Activation of TMEM16A by stimulation of purinergic receptors triggers membrane exocytosis basal mucus secretion in airways and intestine (Benedetto et al., 2019a).

Airways that lack expression of TMEM16A demonstrate goblet cell metaplasia and mucus accumulation (Benedetto et al., 2019a; Cabrita et al., 2019; He et al., 2020). A shift of basal progenitors toward differentiation into the secretory lineage was shown to be TMEM16A dependent (He et al., 2020). Cell specific knockout of TMEM16A and TMEM16F in FoxJ1-positive ciliated airway epithelial cells also caused goblet cell metaplasia (Benedetto et al., 2019a; Cabrita et al., 2019). We hypothesized that release of a signal molecule that normally restricts transdifferentiation into secretory cells is compromised in the absence of TMEM16A/F. Gupta et al. (2019) reported an intercellular communication between airway epithelial cells that is mediated by exosome-like vesicles, and Gomi and coworkers demonstrated JAG1-mediated Notch signaling as a regulator of secretory cell differentiation in the human airway epithelium (Gomi et al., 2016).

Primary airway epithelial cells in an air liquid interface (ALI) culture can be differentiated to develop structural and functional signatures of naive airway epithelia (Yankaskas et al., 1985; Widdicombe, 1990; Cozens et al., 1994; Fulcher et al., 2005; Rayner et al., 2019; Bukowy-Bieryłło et al., 2022). Airway epithelial cells derived from nasal brushings can be expanded in culture and differentiated by growing on permeable supports, often in the presence with proprietary PneumaCult™-ALI media (Rayner et al., 2020; Awatade et al., 2021; Bukowy-Bieryłło et al., 2022). In the present study we used highly differentiated BCi-NS1.1 airway epithelial cells grown in PneumaCult™-ALI media and TMEM16A overexpressing cells to examine the role of TMEM16A/F for airway epithelial differentiation, which may occur by release of extracellular vesicles and Notch signaling. The data support the role of TMEM16A/F for exocytosis and release of extracellular vesicles (which may belong to different types such as exosomes or ectosomes (Mathieu et al., 2021), but do not provide evidence for inhibition of Notch mediated differentiation towards secretory airway cells (He et al., 2017).

## Results

**FIGURE 1:**
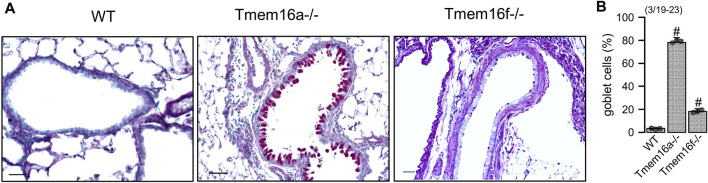
Knockout of Tmem16a and Tmem16f causes goblet cell metaplasia in airway epithelial cells. **(A)** PAS staining of small airways from wild type (WT) mice, and mice with airway ciliated-cell specific knockout of Tmem16a (Tmem16a−/−) and Tmem16f (Tmem16f−/−). Note the pronounced and mild goblet cell metaplasia in Tmem16a knockout and Tmem16f knockout mice, respectively. Bars = 50 µm. **(B)** Percentage of mucus containing (goblet) cells in airways of WT, Tmem16a−/−, and Tmem16f−/− mice, respectively. Mean ± SEM (numbers in parenthesis: number of mice used/number of airways analyzed). ^#^Statistically significant difference when compared to WT (*p* < 0.05; ANOVA).

**FIGURE 2:**
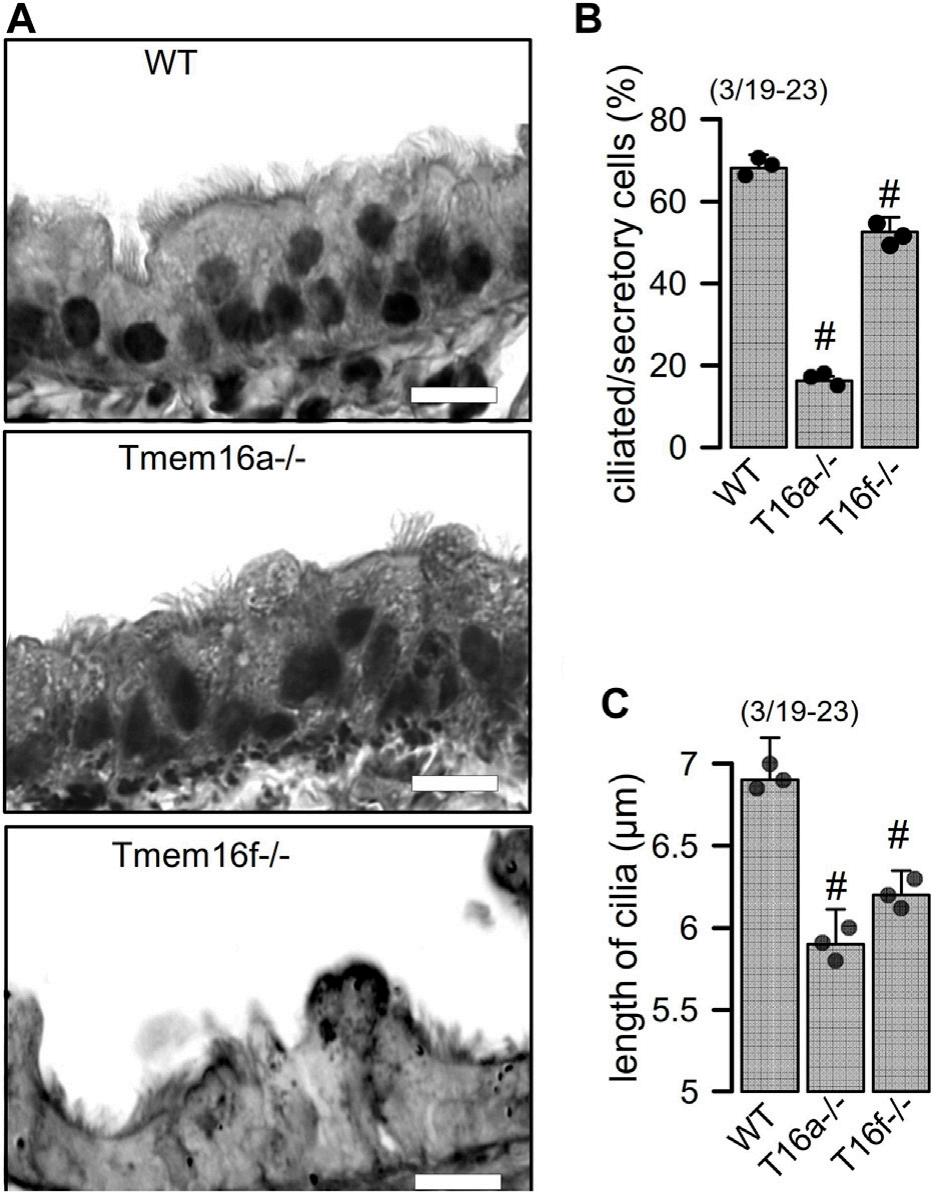
Reduced number of ciliated cells and length of cilia in airways of Tmem16a−/− and Tmem16f−/− mice. **(A)** Airways from WT, Tmem16a−/−, and Tmem16f−/− mice. Bars = 20 µm. **(B)** Percentage of ciliated cells in WT, Tmem16a−/−, and Tmem16f−/− airways, respectively. **(C)** Length of cilia in WT, Tmem16a−/−, and Tmem16f−/− airways, respectively. Mean ± SEM Mean ± SEM (numbers in parenthesis: number of mice used/number of airways analyzed). ^#^Statistically significant difference when compared to WT (*p* < 0.05; ANOVA).

*Knockout of Tmem16a and Tmem16f induces goblet cell metaplasia in airway epithelial cells*. Along with other TMEM16 proteins the Ca^2+^ activated Cl^−^ channel TMEM16A and the phospholipid scramblase TMEM16F have been shown to be expressed in airways and alveoli (Kunzelmann et al., 2012). While small airways of wildtype mice show only few mucus producing cells, pronounced goblet cell metaplasia was observed in airways of mice with cell-specific knockout of TMEM16A in ciliated epithelial cells [FOXJ1-Cre–Tmem16a^flox/flox(+/+)^]. Mucus accumulation was also observed in TMEM16F knockout mice [FOXJ1-Cre–Tmem16f^flox/flox(+/+)^] mice, albeit at a clearly lower level (Figures 1A, B). In high resolution images we analyzed the ratio of ciliated to non-ciliated secretory cells, which was reduced in Tmem16a−/− and Tmem16f−/− mice (Figures 2A, B). Moreover, cilia appeared irregular and the length of cilia was reduced in the knockout animals, which corresponds to previous observations (Figures 2A, C) (He et al., 2017).

**FIGURE 3:**
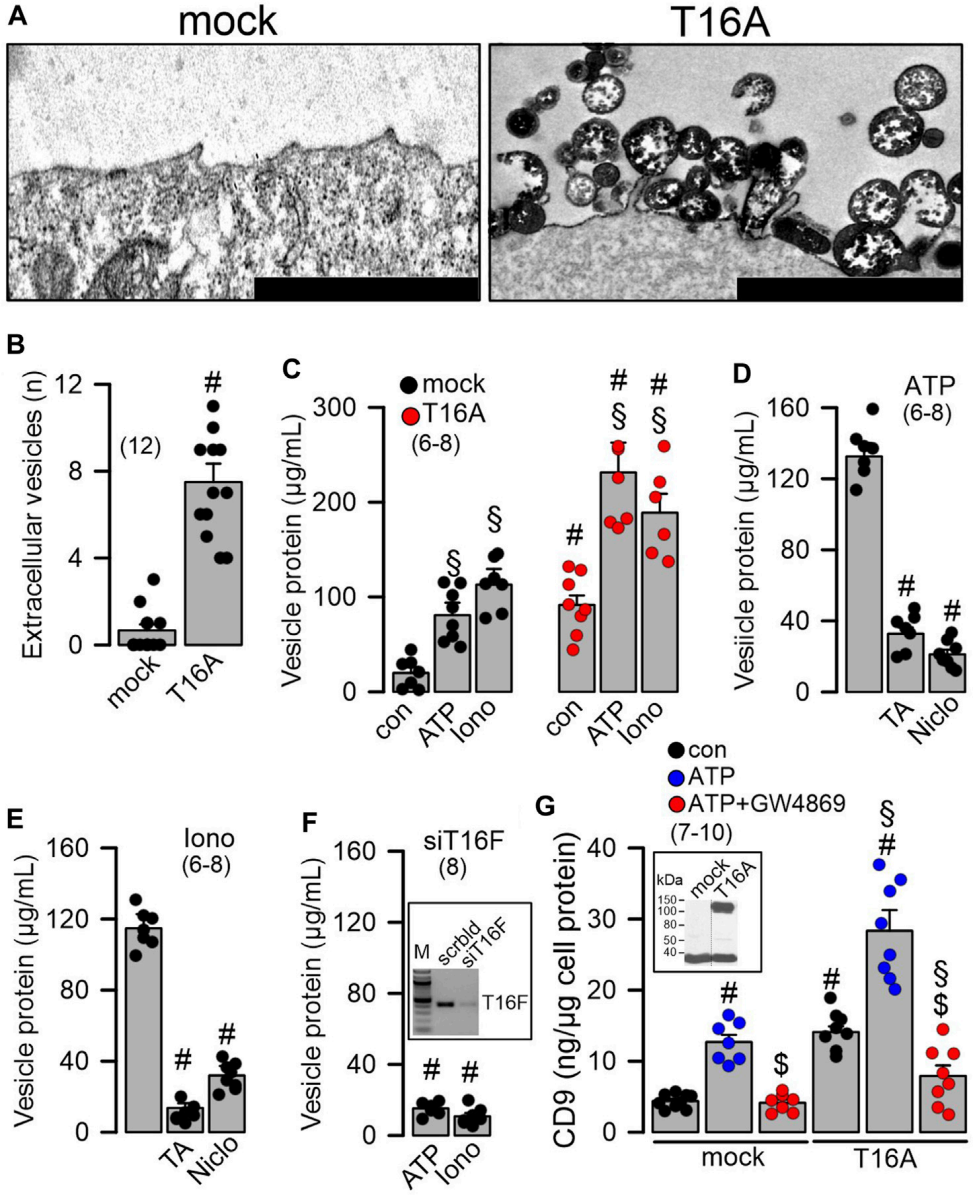
Overexpression of TMEM16A causes formation of extracellular vesicles and release of proteins. **(A)** Formation and release of extracellular vesicles detected in Cos7 cells overexpressing tetracysteine-tagged TMEM16A (T16A). Dark precipitations in plasma membrane and in extracellular vesicles are due to UV-modified electron-dense tetracysteine. Bars indicate 300 nm. **(B)** Number of extracellular vesicles detected in mock-transfected cells and cells overexpressing TMEM16A (T16A). **(C)** Quantification of vesicular proteins released from mock transfected HEK293 cells, and cells overexpressing TMEM16A (T16A) under control conditions and after stimulation with ATP (100 µM) or ionomycin (Iono; 1 µM). **(D)** Release of vesicular protein after stimulation with ATP (100 µM) and inhibition by tannic acid (TA; 3 µM) or niclosamide (Niclo; 0.5 µM). **(E)** Release of vesicular protein after stimulation with ionomycin (Iono; 1 µM) and inhibition by tannic acid (TA; 3 µM) or niclosamide (Niclo; 0.5 µM). **(F)** Release of vesicular proteins induced by ATP or Iono from cells after siRNA-knockdown of endogenous TMEM16F. Semiquantitative RT-PCR indicating knockdown of endogenous TMEM16F by siRNA (inset). **(G)** Detection of the vesicular marker CD9 in vesicular proteins released upon stimulation with ATP (100 µM) in mock-transfected and TMEM16A overexpressing cells. Western demonstrating overexpression of TMEM16A in HEK293 cells (inset). Inhibition of released CD9 by the sphingomyelinase inhibitor GW4869 (5 µM/24 h). Mean ± SEM (number of experiments). ^#^Statistically significant difference when compared to mock and con, respectively (*p* < 0.05; ANOVA). ^§^Statistically significant difference when compared to con (*p* < 0.05; ANOVA). ^$^Significant effect of GW4869 (*p* < 0.05; unpaired *t*-test).

*TMEM16A and TMEM16F facilitate release of extracellular vesicles.* We asked how loss of expression of TMEM16 proteins in ciliated cells causes an increase in the number of secretory (club and goblet) cells, and whether this may be related to vesicular and/or Notch signaling (Sheldon et al., 2010; Rock et al., 2011; Gupta et al., 2019). To that end we overexpressed TMEM16A in Cos7 cells and analyzed formation of extracellular vesicles using electron microscopic images. The results demonstrated that cells expressing TMEM16A produce large numbers of extracellular vesicles, while mock-transfected cells only occasionally produce extracellular vesicles (Figures 3A, B; Supplementary Figure S6). We isolated supernatant and measured protein released from mock-transfected and TMEM16A-overexpressing cells. Protein was isolated from cells under control conditions and after stimulation with ATP or ionomycin (Iono), which both are known to increase intracellular Ca^2+^. In mock-transfected cells, the release of protein (termed vesicular protein) was low under control (basal) conditions and was augmented by stimulation with ATP or Iono (Figure 3C). Basal release was enhanced in TMEM16A-expressing cells when compared to mock-transfected cells (Figure 3C). Moreover, protein release stimulated by ATP or Iono was larger in TMEM16A-expressing cells. The release of vesicular protein by ATP or Iono was strongly inhibited by tannic acid (5 µM) and niclosamide (1 µM), which are both inhibitors of TMEM16A and TMEM16F (Figures 3D, E). In contrast to TMEM16A, endogenous expression of TMEM16F is detected in HEK293 cells. We transfected siRNA-TMEM16F which strongly attenuated expression of TMEM16F by 91.45% ± 3.7% (*n* = 4) (c.f. inset Figure 3F). Suppression of TMEM16F strongly inhibited the release of vesicular protein that was stimulated by ATP or Iono (Figure 3F). To further validate TMEM16A-dependent release of extracellular vesicles, we analyzed the appearance of the vesicular marker protein CD9 in the supernatant. ATP stimulated the release of CD9 and expression of TMEM16A further enhanced release of CD9 (Figure 3G). GW4869, the inhibitor of biogenesis and release of extracellular vesicles (Essandoh et al., 2015), strongly inhibited ATP-induced release of CD9. Inhibition of CD9-release by GW4869 was more pronounced in TMEM16A-expressing cells (Figure 3G). Taken together the data suggest a role to TMEM16A/F for formation and/or release of membrane vesicles, which could be related to goblet cell metaplasia in the airways of TMEM16A/F-knockout mice. Extracellular vesicles released from highly differentiated BCi-NS1.1 airway cultures could not be analyzed due to the abundance of ciliated airway epithelial cells. However, preliminary results from proteome analysis of extracellular vesicles released from CFBE human airway epithelial cells and Calu3 human airway submucosal cells show release CD9. We therefore treated Calu3 cells with scrambled RNA or siRNA for TMEM16A or TMEM16F. Vesicle release was inhibited below basal levels (absence of ATPγ-S) by knocking down TMEM16A using siRNA (Supplementary Figure S7). ATP-γ-S induced vesicular release was not inhibited by knock-down of TMEM16F, which is probably explained by the very low expression of TMEM16F in Calu3 cells.

**FIGURE 4:**
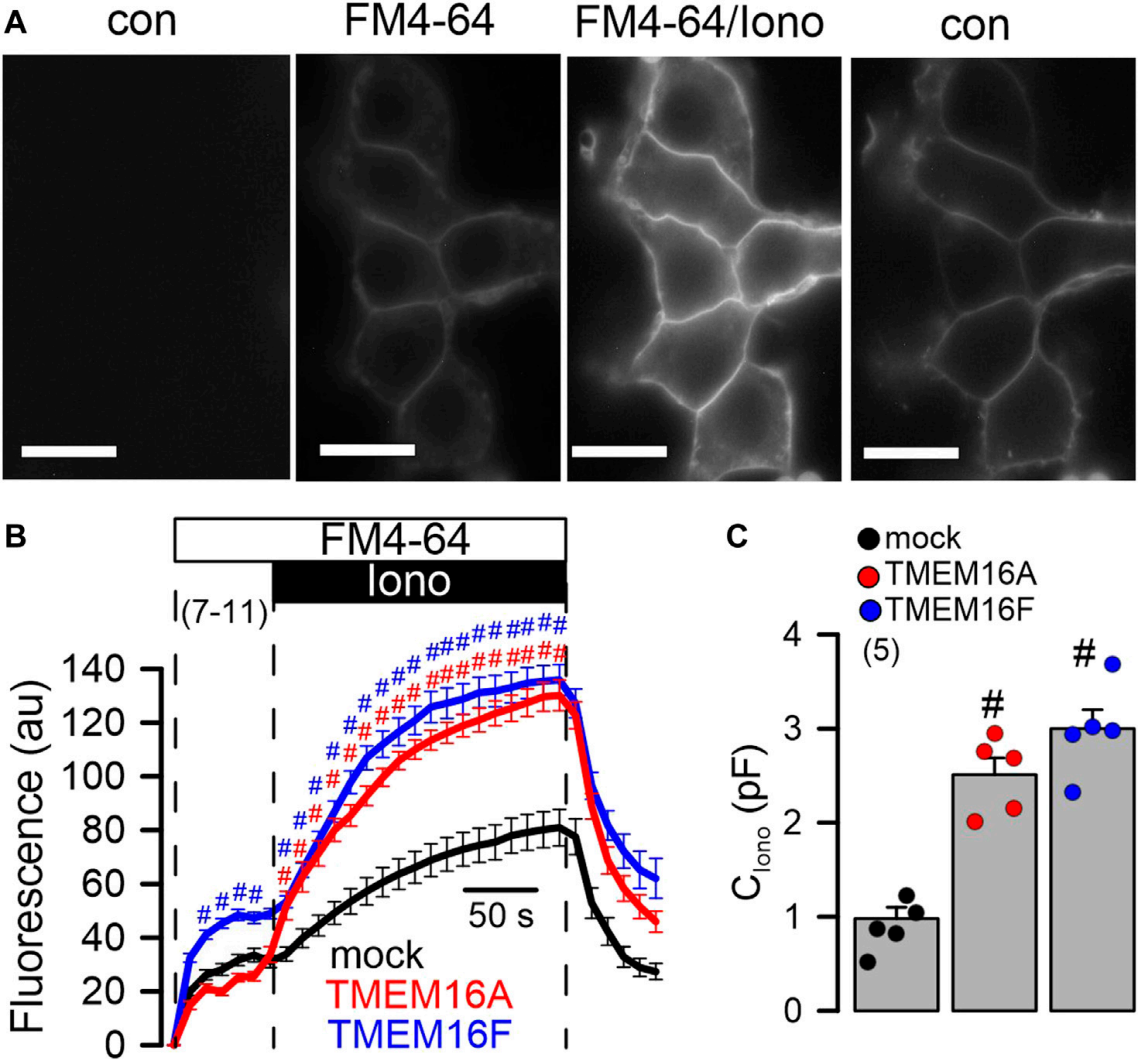
Enhanced exocytosis in HEK293 cells overexpressing TMEM16A or TMEM16F. **(A)** Plasma membrane staining of HEK293 cells overexpressing TMEM16A, using extracellular application of the lipid dye FM4-64. FM4-64 was applied under control conditions (con, absence of FM4-64), after application of FM4-64 (FM4-64), after additional application of ionomycin (1 μM; FM4-64/Iono), and after removal of FM4-64/Iono (con). Bars = 20 µm. **(B)** Time course for FM4-64 fluorescence in cells overexpressing TMEM16A and TMEM16F, and in mock transfected cells. **(C)** Summary of the increase in membrane capacitance **(C)** by ionomycin (1 µM). Mean ± SEM (number of experiments). ^#^Statistically significant difference when compared to mock (*p* < 0.05; ANOVA).

*Cellular exocytosis is supported by TMEM16A and TMEM16F*. Exocytosis and release of proteins requires increase in intracellular Ca^2+^ (Olivero et al., 2021). Previous reports pointed out to the role of TMEM16 proteins for the regulation of intracellular Ca^2+^ and mucus secretion (Jin et al., 2016; Cabrita et al., 2017; Benedetto et al., 2019a). Here we examined membrane exocytosis in HEK293 cells by applying the lipid dye FM4-64 (Benedetto et al., 2016). Application of FM4-64 to the extracellular bath solution inserted into the plasma membrane and caused a slight increase in membrane fluorescence that was further augmented by stimulation with Iono (Figure 4A). The increase in Ca^2+^- (Iono) stimulated fluorescence of the plasma membrane was enhanced in cells expressing TMEM16A or TMEM16F (Figure 4B). The results suggest an increase in Ca^2+^-stimulated exocytosis by TMEM16A/F, which was further confirmed by comparing in patch clamp experiments the ionomycin-induced increase in membrane capacitance in mock, TMEM16A- and TMEM16F-expressing cells (Figure 4C).

**FIGURE 5:**
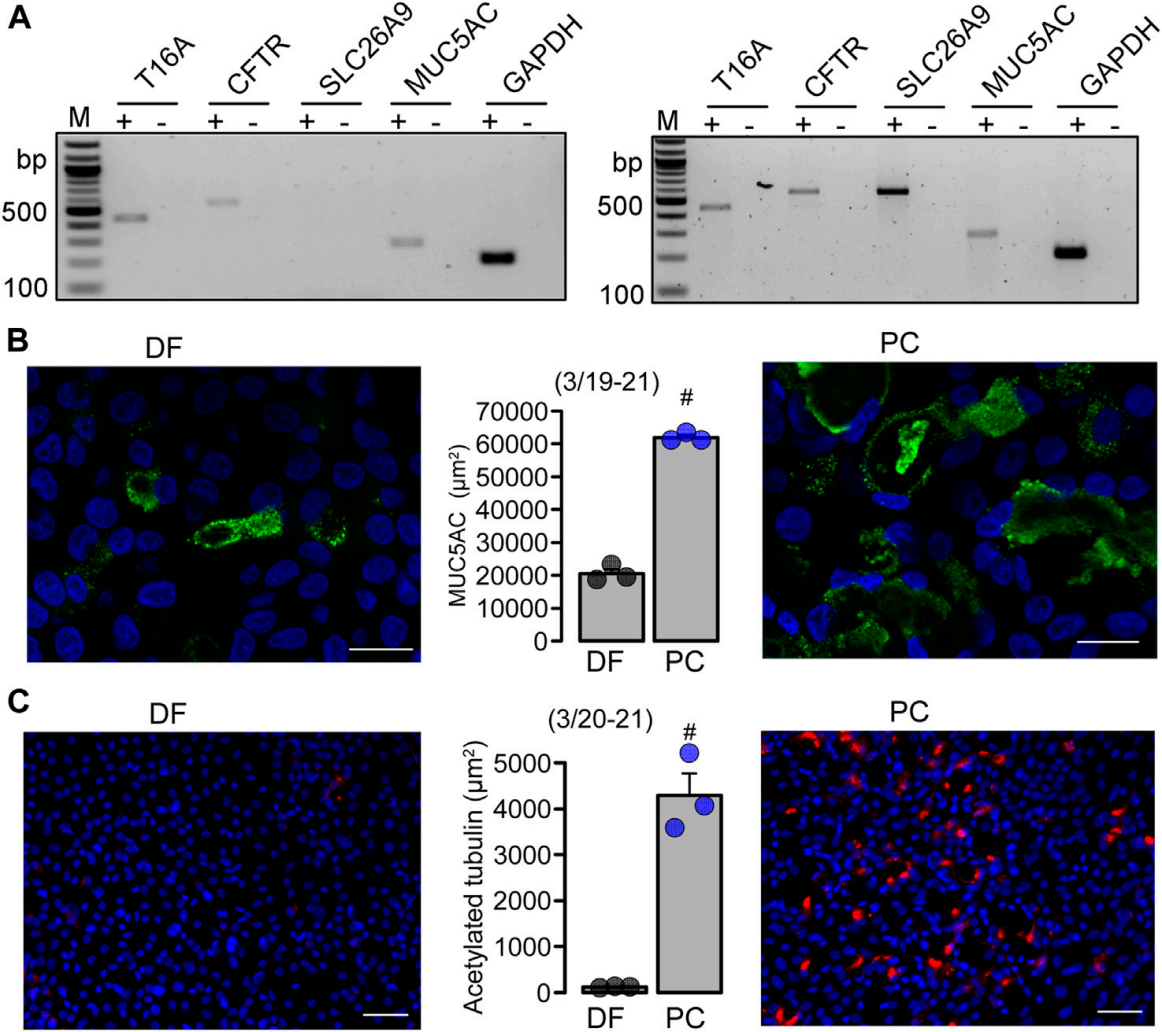
*B*Ci-NS1.1 form a pseudostratified ciliated columnar epithelium when grown under PneumaCult^TM^-ALI conditions. **(A)** Expression of epithelial Cl^−^ channels and MUC5AC in DF and PC cultures. **(B, C)** Immunocytochemistry of MUC5AC (Bars = 20 µm) and acetylated tubulin (Bars = 50 µm) and quantitative analysis in BCi-NS1.1 cells grown as ALI culture in differentiation media or PneumaCult™ media. Mean ± SEM (number of experiments). ^#^statistical difference when compared to DF (*p* < 0.05; unpaired *t*-test).

*Differentiation into a ciliated airway epithelium does not require the function of TMEM16A or TMEM16F.* We further elucidated the role of TMEM16A/F for goblet cell metaplasia in BCi-NS1.1 human basal airway epithelial cells. These progenitor cells are capable of differentiating into secretory cells when grown in standard differentiation media (DF) on permeable supports under an air liquid interface (ALI) (Gomi et al., 2016) (Supplementary Figure S1). However, when using PneumaCult™ (PC) media, differentiation into a pseudostratified ciliated columnar epithelium containing numerous secretory cells, was largely enhanced (Figure 5; Supplementary Figures S2, S3). Secretory Cl^−^ channels such as CFTR and TMEM16A, and the Cl^−^ transporter SLC26A9 were found to be expressed in differentiated BCi-NS1.1 airway cultures grown in DF and PC media, along with expression of MUC5AC (Figure 5; Supplementary Figure S3). Moreover, airway cells grown in PC media demonstrated ion transport properties that were similar to those observed in the native human airway epithelium (Supplementary Figure S2).

Expression of the transcriptional regulators FoxJ1 (forkhead box J1; ciliated cells), CFAP53 (cilia and flagella associated protein 53; ciliated cells), CFAP276 (cilia and flagella associated protein 276; ciliated cells), and FoxI1 (forkhead Box I1; ionocytes) was strongly upregulated in cells grown in PC media. Moreover, expression of transcriptional regulators was not affected by niclosamide, suggesting that a Cl- transporting function of TMEM16A or TMEM16F is not required for differentiation towards a ciliated airway epithelium (Supplementary Figure S2). Importantly, expression of TMEM16A was large in non-differentiated (plastic grown) BCI-NS1.1 cells but was reduced in polarized cells, similar to earlier observations (Simoes et al., 2019) (Supplementary Figure S3). In contrast, expression of the regulator of TMEM16A, CLCA1, was upregulated in parallel with downregulation of TMEM16A (Supplementary Figure S3). We compared expression of major epithelial ion channels when BCi-NS1.1 ALI-cultures were grown either in DF or PC media. Moreover, we analyzed whether expression was affected by additional exposure to the interleukin IL-13. Ion channels supporting Cl^−^ secretion such as CFTR and TMEM16A, as well as the Ca^2+^ activated K^+^ channel KCNN4 were strongly upregulated in PC media, while SLC26A9, TMEM16F, ßENaC and CLCA1 were not affected (Supplementary Figure S3B). IL-13 slightly further enhanced expression of TMEM16A and CFTR, but significantly upregulated CLCA1. The results indicate that PneumaCult™ medium induces a strongly proliferative and Cl^−^/mucus-secretory phenotype that appears to have inflammatory properties.

**FIGURE 6:**
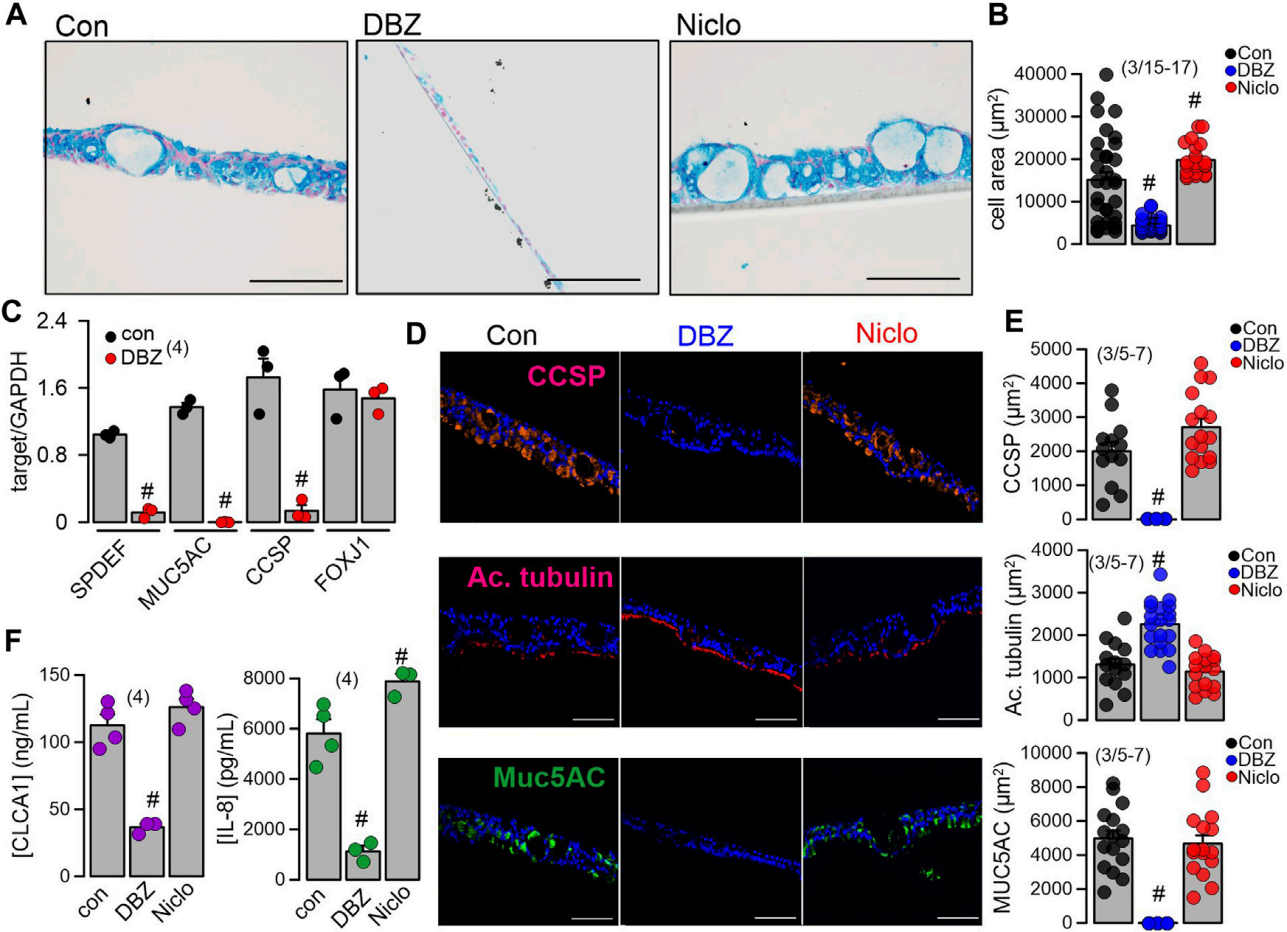
Inhibition of γ-secretase inhibits mucoid hyperplasia of BCi-NS1 human airway epithelial cells. **(A)** Inhibition of the mucosecretory phenotype and hyperplasia in differentiated BCi-NS1 cells grown for 30 days in PneumaCult™-ALI media, by the γ-secretase inhibitor DBZ (50 nM/17 days), but not by niclosamide (Niclo, 200 nM) which even further enhanced hyperplasia. Bars = 100 µm. **(B)** Effects of DBZ and Niclo on tissue hyperplasia. **(C)** RT-PCR analysis of the expression of SPDEF, MUC5AC, CCSP, and FoxJ1 and the effect of DBZ. **(D, E)** Immunocytochemistry of acetylated tubulin, MUC5AC, and CCSP in BCi-NS1 cells in the absence or presence of DBZ and quantitative analysis. **(F)** Release of the cytokines CLCA1 and IL-8 from BCi-NS1 cells grown in ALI cultures in the absence or presence of DBZ (50 nM/17 days) and Niclo (200 nM/17 days). Mean ± SEM (number of experiments). ^#^Statistically significant difference when compared to con (*p* < 0.05; ANOVA).

*Differentiation into a mucosecretory phenotype depends on Notch signaling.* JAG1-dependent Notch-signaling was found to be required for differentiation of basal cells into secretory cells (Gomi et al., 2016). Anti-JAG1/2 antibodies caused a near complete loss of airway goblet cells in mice (Lafkas et al., 2015). The gamma-secretase is essential for Notch signaling because it cleaves the Notch intracellular domain. We inhibited the γ-secretase by dibenzazepine (DBZ) and observed a pronounced dedifferentiation and loss of height of the BCi-NS1.1 airway epithelium (Figures 6A, B). In contrast, the inhibitor of TMEM16A/F, niclosamide, further enhanced mucus staining and accumulation of mucus-filled and empty vacuoles (Figures 6A, B). Impressively, treatment with DBZ eliminated expression of the transcriptional factor and integrator of goblet cell differentiation SPDEF (SAM-pointed domain–containing ETS-like factor) (Figure 6C).

**FIGURE 7:**
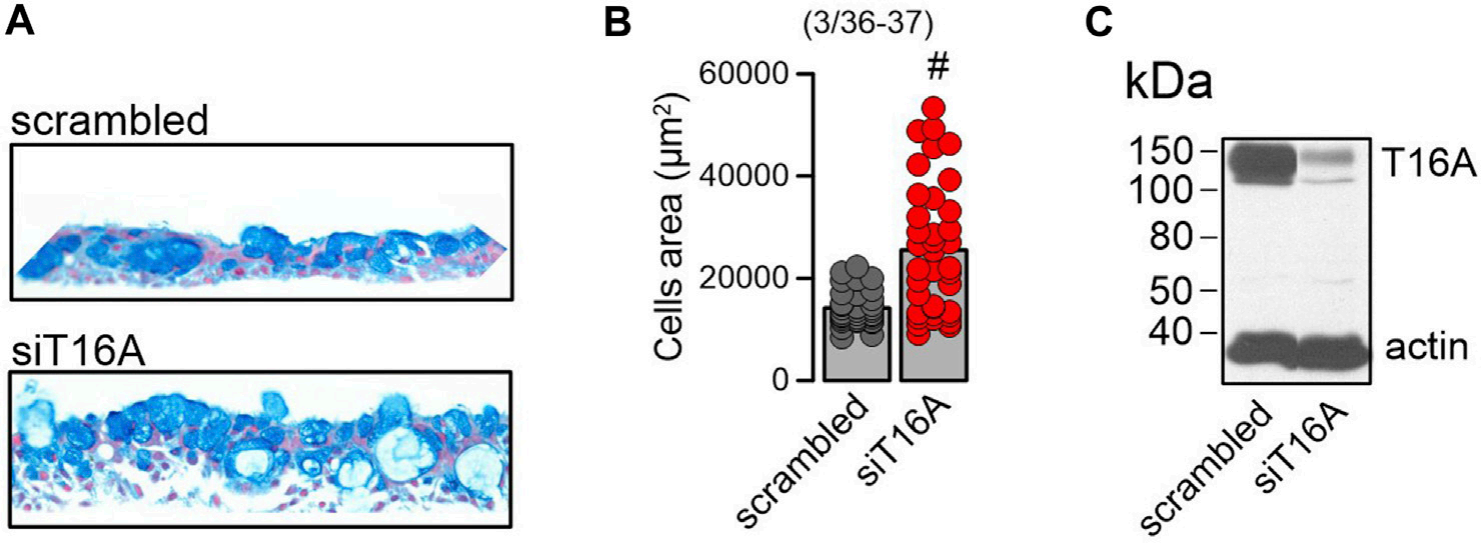
Lack of TMEM16A expression augments *mucoid hyperplasia*. **(A)** Increase of the mucosecretory phenotype and hyperplasia by knockdown of TMEM16A expression in differentiated BCi-NS1.1 cells. **(B)** Quantitative analysis of the increase in cell area by knockdown of TMEM16A. **(C)** Western of TMEM16A demonstrating knockdown in BCi-NS1.1 cells. Mean ± SEM (number of experiments). ^#^Statistically significant difference when compared to con (*p* < 0.05; paired *t*-test).

Possibly as a consequence of suppression of SPDEF, expression of MUC5AC and CCSP were also eliminated by DBZ, while expression of FOXJ1 was unaffected (Figure 6C). We also examined expression of CCSP (club cells), acetylated tubulin (ciliated cells), and MUC5AC (goblet cells) in polarized BCi-NS1.1 airways using immunocytochemistry. Expression of CCSP and MUC5AC was completely suppressed by DBZ, while the number of ciliated airway epithelial cells was increased (Figures 6D, E). In contrast, niclosamide had no effect on expression of CCSP, MUC5AC or acetylated tubulin. Notably, DBZ also inhibited the release of inflammatory cytokines CLCA1 and IL-8, in contrast to niclosamide, which did not inhibit cytokine release (Figure 6F). Taken together Notch signaling is essential for goblet cells metaplasia and development of a mucosecretory airway epithelium. In such an airway epithelium transformed by pronounced activation of Notch-signaling, inhibition of TMEM16A by niclosamide or siRNA-knockdown of TMEM16A-expression did not reduce mucus production, but appears to inhibit mucus release leading to expansion of mucus-filled goblet cells (Figure 7A).

## Discussion

*Activation of Notch signaling in BCi-NS1.1 airway epithelial cells activates Cl* ^*−*^ *and mucus secretory pathways.* PneumaCult™-ALI medium is widely used to induce differentiation of cultured airway epithelia (Rayner et al., 2019; Leung et al., 2020; Awatade et al., 2021; Bukowy-Bieryłło et al., 2022). We found a pronounced upregulation of proliferation and differentiation into a mucus- and Cl^−^-secreting ciliated airway epithelium by PneumaCult™-ALI medium. Unfortunately the composition of PneumaCult™-ALI medium is not released, but our present data suggest a pronounced activation of Notch-signaling, as inhibition of γ-secretase largely inhibited goblet cell metaplasia and the presence of ciliated cells. Earlier studies demonstrated pronounced inhibition of mucus production *in vitro* and *in vivo* by inhibitors of TMEM16A and by knockdown TMEM16A expression (Cabrita et al., 2020; Centeio et al., 2021a; Ousingsawat et al., 2022a). In the presence of PneumaCult™-ALI medium and activation of Notch-signaling, inhibition of expression (siRNA) or function (niclosamide) of TMEM16A did not reduce mucus production, but appeared to inhibit mucus release leading to expansion of mucus-filled goblet cells, similar to earlier observations (Benedetto et al., 2019a; Cabrita et al., 2019) (Figures 6, 7). We also examined whether other factors may participate in the airway epithelial differentiation induced by PneumaCult™-ALI. However, inhibition of IL-8 receptors by reparixin (500 nM), the metalloproteinase inhibitor marimastat (150 nM) or the STAT-3 inhibitor BP-1-102 (100–1,000 nM) did not show any effects (Supplementary Figures S4, S5). Thus, Notch signaling is probably the most essential mechanism by which club/goblet cell differentiation is controlled.

*Notch signaling is inducing secretory cell differentiation in BCi-NS1.1 cells.* Jag1 and Notch4 have been implicated earlier in allergic asthma and inflammation (Xia et al., 2018). Epithelial Notch signaling also regulates lung alveolar morphogenesis and airway epithelial integrity (Tsao et al., 2016). Another study showed a role of Notch signaling in preventing mucous metaplasia in mouse conducting airways during postnatal development (Tsao et al., 2011). Lafkas et al. (2015) suggested the use of therapeutic anti-Jag1/anti-Jag2 antibodies to prevent Notch-dependent trans-differentiation into secretory airways. Gomi et al. (2015) demonstrated that activation of Notch1 or Notch3 signaling shifts human airway basal cell differentiation toward the secretory pathway. Notch3-Jagged signaling controls the pool of undifferentiated airway progenitors (Mori et al., 2015), while Notch2 is required for inflammatory cytokine-driven goblet cell metaplasia in the lung (Danahay et al., 2015). Morimoto and coworkers proposed that different assemblies of Notch receptors coordinate the distribution of the major bronchial Clara, ciliated and neuroendocrine cells (Morimoto et al., 2012) and Rock et al. (2011) demonstrated that Notch-activation is greatly enhanced during tissue repair.

*Lack of TMEM16A induces goblet cell metaplasia.* He and coworkers reported airway differentiation towards secretory cells in airways of TMEM16A-knockout animals (He et al., 2020). They observed cilia-secretory hybrid cells in postnatal airways and early signs of inflammation. It was suggested that under physiological conditions the presence of TMEM16A inhibits differentiation towards secretory cells (He et al., 2020). Possibly cytosplasmic Cl^−^ concentrations couple to epithelial morphogenesis (He et al., 2017). We also observed signs of goblet cell metaplasia in airways of mice with a ciliated-cell specific knockout of TMEM16A. Based on our experiments we interpreted these results as a defect in constitutive mucus secretion with consecutive increase in cellular mucus content (Benedetto et al., 2019a). Similarly, knockout of TMEM16F showed a similar, albeit milder phenotype (Cabrita et al., 2019). Moreover, we observed enhanced mucus content in intestinal goblet cells in animals with intestinal-epithelial specific knockout of TMEM16A or TMEM16F. Mucus was released from intestinal goblet cells by stimulation of luminal purinergic receptors with ATP, which was compromised in mice lacking intestinal epithelial expression of TMEM16A or TMEM16F. Notably, mucus release was completely abolished by niclosamide and other TMEM16-inhibitors (Benedetto et al., 2019a; Cabrita et al., 2019).

*Compromised release and accumulation of mucus in airways lacking expression of TMEM16A/F.* These and other data demonstrate the crucial role of TMEM16A/F for exocytosis and insertion of ion channels into the plasma membrane (Benedetto et al., 2019b; Park et al., 2020; Schreiber et al., 2022). The Hilgemann team demonstrated TMEM16F-dependent large-scale surface membrane expansion leading to opening of plasma membrane compartments and extensive shedding of ectosomes (Bricogne et al., 2019; Deisl et al., 2021). Finally, we showed earlier that TMEM16A activates TMEM16F and supports scrambling by enhancing compartmentalized submembraneous Ca^2+^ levels (Cabrita et al., 2017; Schreiber et al., 2018; Ousingsawat et al., 2019), super-resolution microscopy demonstrated colocalization of TMEM16F and the TMEM16A paralogue TMEM16B in discrete microdomains which strongly augments ATP-activated whole cell Cl^−^ currents (Henkel et al., 2015).

Taken together the available data demonstrate the role of TMEM16A/F for exocytosis and formation of extracellular vesicles. In the absence of these TMEM16 paralogues, mucus release appears to be inhibited. We hypothesize that liberation of yet unknown factors that inhibit differentiation into a secretory epithelium may be compromised.

## Materials and methods

### Animals

All animal experiments were approved by the local ethics committee of the Government of Unterfranken/Würzburg (AZ: 55.2-2532-2-677) and were conducted according to the guidelines of the American Physiologic Society and the German law for the welfare of animals. Generation of mice with selective knockout of Tmem16a and Tmem16f in ciliated airway epithelial cells (FOXJ1-Cre–Tmem16a^flox/flox^; FOXJ1-Cre–Tmem16f^flox/flox^) and intestinal epithelial cells (Vil1-Cre–Tmem16a^flox/flox^; Vil1-Cre–Tmem16f^flox/flox^) and genotyping of these animals has been reported earlier (Benedetto et al., 2017; Cabrita et al., 2019).

### Cell culture

Human embryonic kidney HEK293 stably expressing eYFP-I152L (HEK-YFP) or co-expressing TMEM16A and eYFP-I152L (HEK-TMEM16A-YFP) were grown in DMEM low glucose medium supplemented with 10% (v/v) exosome depleted FBS, 1% (v/v) L-glutamine 200 mM and 10 mM HEPES (all from Capricorn Scientific, Ebsdorfergrund, Germany). BCi-NS1.1 Cos-7 cells were grown as described earlier (Tian et al., 2011). ALI cultures grown in differentiation (DF) medium or PneumaCult™-ALI medium as described earlier (Centeio et al., 2021b; Ousingsawat et al., 2022b).

### ReAsH labeling and electron microscopy

Transfection of cells, labeling with resorufin arsenical hairpin binding reagent (ReAsH; Invitrogen) and EM 902 transmission electron microscopy has been described in an earlier study (Tian et al., 2011).

### Isolation and quantification of vesicular protein

HEK293 parental and overexpressing TMEM16A were grown to 70% confluency, washed 3 times with PBS and incubated for 24 h in serum free media, with 1 µM ATPgammaS or 1 µM ionomycin. The media was collected after 24 h and proceeded to purification of extracellular vesicles. Purification was performed by ultracentrifugation, as described in Lobb et al. (2015). Briefly, media was centrifuged using at 300 g at 4°C for 10 min to remove detached cells. Supernatant was collected and centrifuged at 2000 *g* at 4°C for 10 min to remove dead cells. The resulting supernatant was filtered through 0.22 mm filters (Merck Millipore) to remove contaminating apoptotic bodies, microvesicles and cell debris. Clarified media was then centrifuged in a Beckman Optima™ MAX-E Ultracentrifuge with the rotor TLA-55 at 100,000 g at 4°C for 70 min to pellet extracellular vesicles. The supernatant was carefully removed, and pellets were resuspended in 1 mL of ice-cold PBS and pooled. A second round of ultracentrifugation (100,000 g at 4°C for 70 min) was carried out, and the resulting pellet lysed in sample buffer containing 50 mM Tris-HCl, 150 mM NaCl, 50 mM Tris, 100 mM dithiothreitol, 1% Nonidet P-40, 0.5% deoxycholate sodium, and 1% protease inhibitor mixture (Sigma, Taufkirchen, Germany). Release of extracellular vesicles was inhibited by tannic acid (TA, 10 µM) or Niclosamide (Niclo, 1 µM). The media was collected after 24 h, and pellets containing extracellular vesicles were lysed in sample buffer containing 50 mM Tris-HCl, 150 mM NaCl, 50 mM Tris, 100 mM dithiothreitol, 1% Nonidet P-40, 0.5% deoxycholate sodium, and 1% protease inhibitor mixture (Sigma, Taufkirchen, Germany). The concentration of total vesicular protein was measured using Bio-Rad protein Assay (Bio-Rad Laboratories, California, United States). For CD9 quantification HEK293 cells were treated with 5 μM GW4869, an inhibitor of extracellular vesicles (Essandoh et al., 2015) for 24 h or 100 µM ATP for 10 min in serum free media. Purified vesicles were quantified using ExoQuant a double sandwich enzyme-linked immunoassay for CD9 (BioVision Incorporated, Milpitas, United States).

### FM 4-64 analysis

HEK293 cells in glass coverslips were mounted in a chamber and continuously perfused at 37°C with Ringer solution containing 2 mg/mL FM 4–64. After 1 min, the cells were stimulated with 1 mM ionomycin (Iono) for 3 min following washout. FM 4-64 was excited at 546 ± 12 nm, and emission was recorded at 575–640 nm (Filter Set 20) using Axio Observer microscope and ZEN software (Carl Zeiss AG, Jena, Germany).

### RT-PCR

For RT-PCR total RNA from tissues or cells were isolated using NucleoSpin RNA II columns (Macherey-Nagel, Düren, Germany). Total RNA (0.5 µg/25 µL reaction) was reverse-transcribed using random primer (Promega, Mannheim, Germany) and M-MLV Reverse Transcriptase RNase H Minus (Promega, Mannheim, Germany). Each RT-PCR reaction contained sense (0.5 µM) and antisense primer (0.5 µM) (Table 1), 0.5 µL cDNA and GoTaq Polymerase (Promega, Mannheim, Germany). After 2 min at 95°C cDNA was amplified (targets 35 cycles, reference GAPDH 25 cycles) for 30 s at 95°C, 30 s at 56°C and 1 min at 72°C. PCR products were visualized by loading on Midori Green Xtra (Nippon Genetics Europe) containing agarose.

**TABLE 1:** Primer (s: sense, as: antisense) for RT-PCR.

Gene accession number	Primer	Size (bp)
human CCSP	s: 5′- TCT​GCT​GCA​GCT​CCG​CTT​C	236
NM_003357.5	as: 5′- CTA​ATT​ACA​CAG​TGA​GCT​TTG​G	
human CFAP53	s: 5′- AGC​TGA​GCA​CCA​TCT​AGA​AAG	439
NM_145020.5	as: 5′- CTC​ACA​CAC​CTT​CTT​CTG​ATG	
human CFAO276	s: 5′- CAA​AGG​ATG​ACC​TGG​ACT​TC	291
NM_001245025.3	as: 5′- CAC​CAT​CTT​TCT​TTC​GGG​AG	
human CFTR	s: 5′- CTC​ATT​AGA​AGG​AGA​TGC​TCC​TG	568
NM_000492.4	as: 5′- GCT​CTT​GTG​GAC​AGT​AAT​ATA​TCG	
human FoxI1	s: 5′- CAC​TCT​CAG​CCA​GAT​CTA​CC	626
NM_012188.5	as: 5′- GCA​CAG​ATC​CTC​CAT​AGC​TG	
human FOXJ1	s: 5′- CAC​GCT​CAT​CTG​CAT​GGC​C	451
NM_001454.4	as: 5′- CGG​CTG​TTT​GCG​CTT​ATG​C	
human MUC5AC	s: 5′- GCT​CAG​CTG​TTC​TCT​GGA​CG	279
NM_001304359.2	as: 5′- GTC​ACA​TTC​CTC​AGC​GAG​GTC	
human SLC26A9	s: 5′- CAT​TTG​CTG​TGC​GCT​TTC​TG	568
NM_052934.4	as: 5′- CCG​CTT​CTC​CTG​CTT​CTT​G	
human SPDEF	s: 5′- GTG​CTC​AAG​GAC​ATC​GAG​AC	423
NM_012391.3	as: 5′- CCT​AAT​GAA​GCG​GCC​ATA​GC	
human TMEM16F	s: 5′- GTA​TTT​GTA​AAA​GTA​CAC​GCA​CC	463
NM_001025356.3	as: 5′- GAG​GAT​GAG​CCC​ATT​CTC​TG	
monkey TMEM16F	s: 5′- GTT​TAC​TTC​ATC​CTC​TCT​CGG	433
XM_028829416.1	as: 5′- GAC​AAA​GCC​TAT​CAC​ACT​GAG	
human TMEM16A	s: 5′- CGA​CTA​CGT​GTA​CAT​TTT​CCG	445
NM_018043.7	as: 5′- GAT​TCC​GAT​GTC​TTT​GGC​TC	
GAPDH NM_001289726	s: 5′- GTA​TTG​GGC​GCC​TGG​TCA​C	200
	as: 5′- CTC​CTG​GAA​GAT​GGT​GAT​GG	

### Immunocytochemistry

BCi-NS1.1 cells grown under polarized conditions in an air liquid interface were fixed in 4% PFA/PBS for 20 min at room temperate and embedded afterwards in paraffin. Staining was performed as described recently (Centeio et al., 2021b). In brief, paraffin slices (5 μm) were deparaffinized, stained with standard Alcian blue and counterstained with Nuclear Fast Red solution (Sigma-Aldrich, St. Louis, Missouri, United States). After dehydration and clearing steps, sections were mounted in DePeX mounting medium (SERVA Electrophoresis, Heidelberg, Germany). Stains were assessed by light microscopy. Mucus-stained (blue) areas were quantified using ImageJ. Immunostaining of airway epithelial cells was described in an earlier report (Jo et al., 2022). Briefly, cells were incubated with rabbit anti-SCL26A9 antibody (1:100, raised against mouse SCL26A9 aa 11-29, DRAAYSLSLFDDEFEKKDR, Davids Biotechnologie, Regensburg, Germany), anti-acetylated tubulin antibody (T7451, Sigma-Aldrich, Germany; 1:100), anti-CCSP antibody (sc-365992, Santa Cruz Biotechnology, Inc., Germany, 1:300), and anti-MUC5AC antibody (ab3649, abcam, Germany, 1:100), respectively. Goat anti-mouse/Alexa Fluor 488 antibody (1:300) or Alexa Fluor 546 antibody (1:400) were used as secondary antibodies. Nuclei were stained with Hoe33342. Immunofluorescence was examined with an Axio Observer microscope equipped with Axiocam 503 mono, ApoTome.2, and ZEN 3.0 (blue edition) software (Zeiss, Oberkochen, Germany).

### Western blotting

Protein was isolated from cells using a lysis buffer containing 25 mM Tris-HCl pH 7.4, 150 mM NaCl, 1 mM EDTA, 5% glycerol, 0.43% Nonidet P-40, 100 mM dithiothreitol (both from PanReac AppliChem, Barcelona, Spain) and 1× protease inhibitor mixture (Roche, Basel, Switzerland). For supernatant protein precipitation, the trichloroacetic acid (TCA) method was used. Proteins were then separated by 8.5% SDS-PAGE and transferred to a PVDF membrane (GE Healthcare, Munich, Germany). Membranes were incubated overnight at 4°C with primary antibodies: rabbit anti-TMEM16A (#ab64085, Abcam, Cambridge, United Kingdom; 1:500), rabbit anti-TMEM16F (PA5-35240, Thermo Fisher Scientific, Waltham, Massachusetts, United States), rabbit anti-human CFTR (#596; Lot: 596TJ10192007, UNC Chapel Hill, United States) rabbit anti-CLCA1 (ab180851, Abcam; 1:1,000), mouse anti-(S)PDEF(G10) (sc-166846, Santa Cruz Biotechnology; 1:250 in 3% (w/v) NFM/TBST-T), rabbit anti-KCNN4 (#APC-064, Alomone Labs, Jerusalem, Israel; 1:500 in 3% (w/v) NFM/TBST-T), rabbit anti-SLC26A9 (NBP2-30425, #P93, Novus, Bio-Techne GmbH, Wiesbaden, Germany), rabbit anti-ßENaC (1:200, #P23, MyBioSource, San Diego, United States), and rabbit anti-Na+/K + -ATPase (sc-28800, Santa Cruz Biotechnology, 1:500). Membranes were incubated afterwards with horseradish peroxidase (HRP)-conjugated goat anti-rabbit or sheep anti-mouse secondary antibodies at room temperature for 2 h and immunoreactive signals were visualized using a SuperSignal HRP Chemiluminescence Substrate detection kit (#34577; Thermo Fisher Scientific, Waltham, Massachusetts, United States).

### Transepithelial using chamber recordings

BCi- NS1.1 human airway epithelial cells polarized on permeable supports were measured under short-circuit conditions in non-perfused chambers with bicarbonate-buffered Ringer solution (mmol/l: NaCl 118.75; KH2PO4 0.4; K2HPO4 1.6; glucose 5; MgSO_4_ 1; Ca-gluconate 1.3, NaHCO_3_ 25; bubbled with 95% O_2_/5% CO_2_). Technical details have been described in previous reports (Benedetto et al., 2020; Jo et al., 2022).

### Capacitance measurements

Membrane capacitance was assessed in fast whole cell patch clamp recordings as described previously (Benedetto et al., 2016). In brief, patch pipettes were filled with a cytosolic-like solution containing KCl 30, K–gluconate 95, NaH_2_PO_4_ 1.2, Na_2_HPO_4_ 4.8, EGTA 1, Ca–gluconate 0.758, MgCl_2_ 1.03, D-glucose 5, ATP 3, pH 7.2. The Ca^2+^ activity was 0.1 μM. Cells grown on coverslips were mounted in a perfused bath chamber on the stage of an inverted microscope (IM35, Zeiss) and kept at 37°C. The bath was perfused continuously with Ringer solution at a rate of 2 mL/min. Further details are described in (Benedetto et al., 2016).

### Materials and statistical analysis

Data are reported as means ± SEM. Student’s *t*-test (for paired or unpaired samples as appropriate) or ANOVA were used for statistical analysis. A value of *p* < 0.05 was accepted as a significant difference.

## Data Availability

The raw data supporting the conclusion of this article will be made available by the authors, without undue reservation.
