# Supplementary material for: TMEM16A/F support exocytosis but do not inhibit Notch-mediated goblet cell metaplasia of BCi-NS1.1 human airway epithelium
Source: Front Physiol. 2023 May 9;14:1157704. doi: 10.3389/fphys.2023.1157704 (PMC10206426; doi:10.3389/fphys.2023.1157704)
Supplement: Supplementary file 1 [file DataSheet7.PDF]

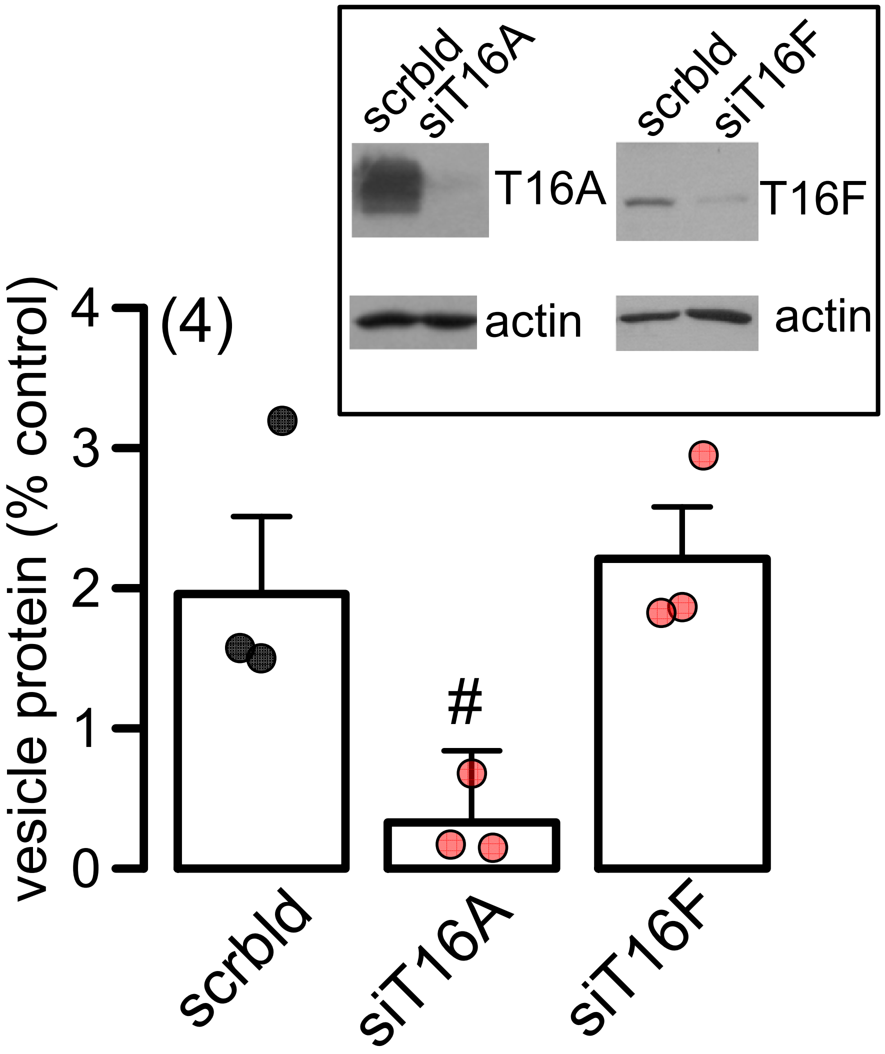

Supplementary Figure 7. *Inhibition of vesicle release from Calu3 human airway epithelial cells by knockdown of TMEM16A.* Vesicle release from Calu-3 cells was induced by stimulation with ATP-γ-S (1 µM, 24 hrs). Cells were treated with scrambled RNA or siRNA for TMEM16A or TMEM16F. Vesicle release was inhibited below basal levels (absence of ATPγ-S) by knocking down TMEM16A using siRNA. ATP-γ-S induced vesicular release was not inhibited by knock-down of TMEM16F. This is probably explained by the very low expression of TMEM16F in Calu3 cells. Mean ± SEM (number of experiments). Inset shows Western blots of TMEM16A and TMEM16F, respectively, in Calu3 cells treated with scrambled RNA (scrbld) or treated with siRNA.
