# Supplementary material for: TMEM16A/F support exocytosis but do not inhibit Notch-mediated goblet cell metaplasia of BCi-NS1.1 human airway epithelium
Source: Front Physiol. 2023 May 9;14:1157704. doi: 10.3389/fphys.2023.1157704 (PMC10206426; doi:10.3389/fphys.2023.1157704)
Supplement: Supplementary file 2 [file DataSheet2.PDF]

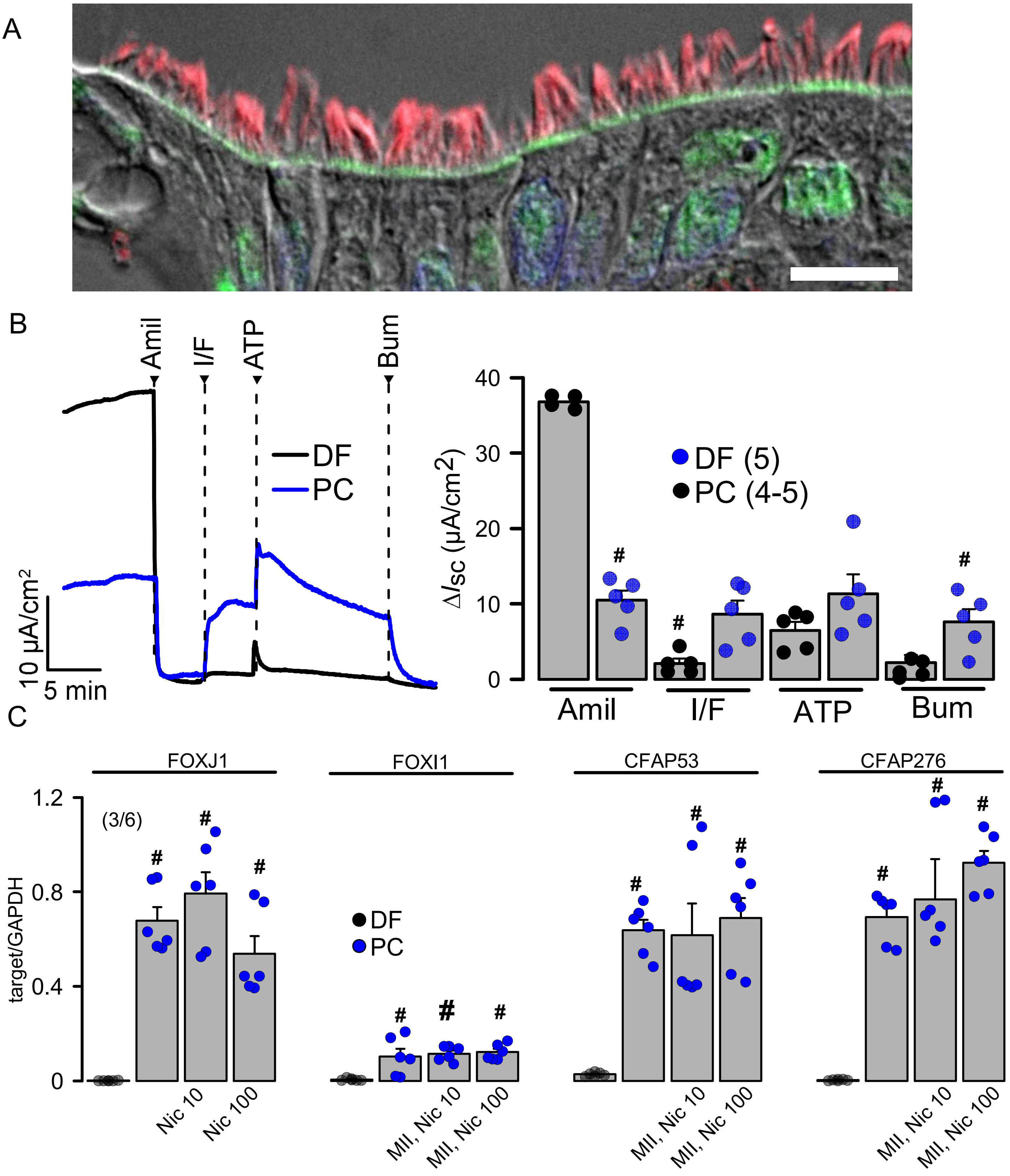

Supplementary Figure 2: *Culture media dependent differentiation of BCi-NS1 human airway epithelial cells.* A) Differentiated BCi-NS1 airway epithelium in ALI/ PneumaCult TM (PC) culture. Acetylated tubulin cilia (red) and SLC26A9 (green) staining. Bar = 20 µm. B) Original recordings and summary of short circuit currents (Isc) showing effects of amiloride (10 µM), IBMX/forskolin (I/F; 100 µM, 2 µM), ATP (100 µM), and bumetanide (Bum; 50 µM) on BCi-NS1 airway epithelia grown in DF (DMEM/Ham's F12 + 2% USG) or PC (PneumaCult TM) media. C) RT-PCR analysis of expression of the ciliated cell and ionocyte markers FoxJ1, FoxI1, CFAP53, and CFAP276 in DF and PC cultures, and the potential effects of niclosamide (Nic, 10 and 100 nM). Mean ± SEM (number of experiments). #statistical difference when compared to DF ($p < 0.05$; unpaired t-test).
