# Supplementary material for: TMEM16A/F support exocytosis but do not inhibit Notch-mediated goblet cell metaplasia of BCi-NS1.1 human airway epithelium
Source: Front Physiol. 2023 May 9;14:1157704. doi: 10.3389/fphys.2023.1157704 (PMC10206426; doi:10.3389/fphys.2023.1157704)
Supplement: Supplementary file 3 [file DataSheet4.PDF]

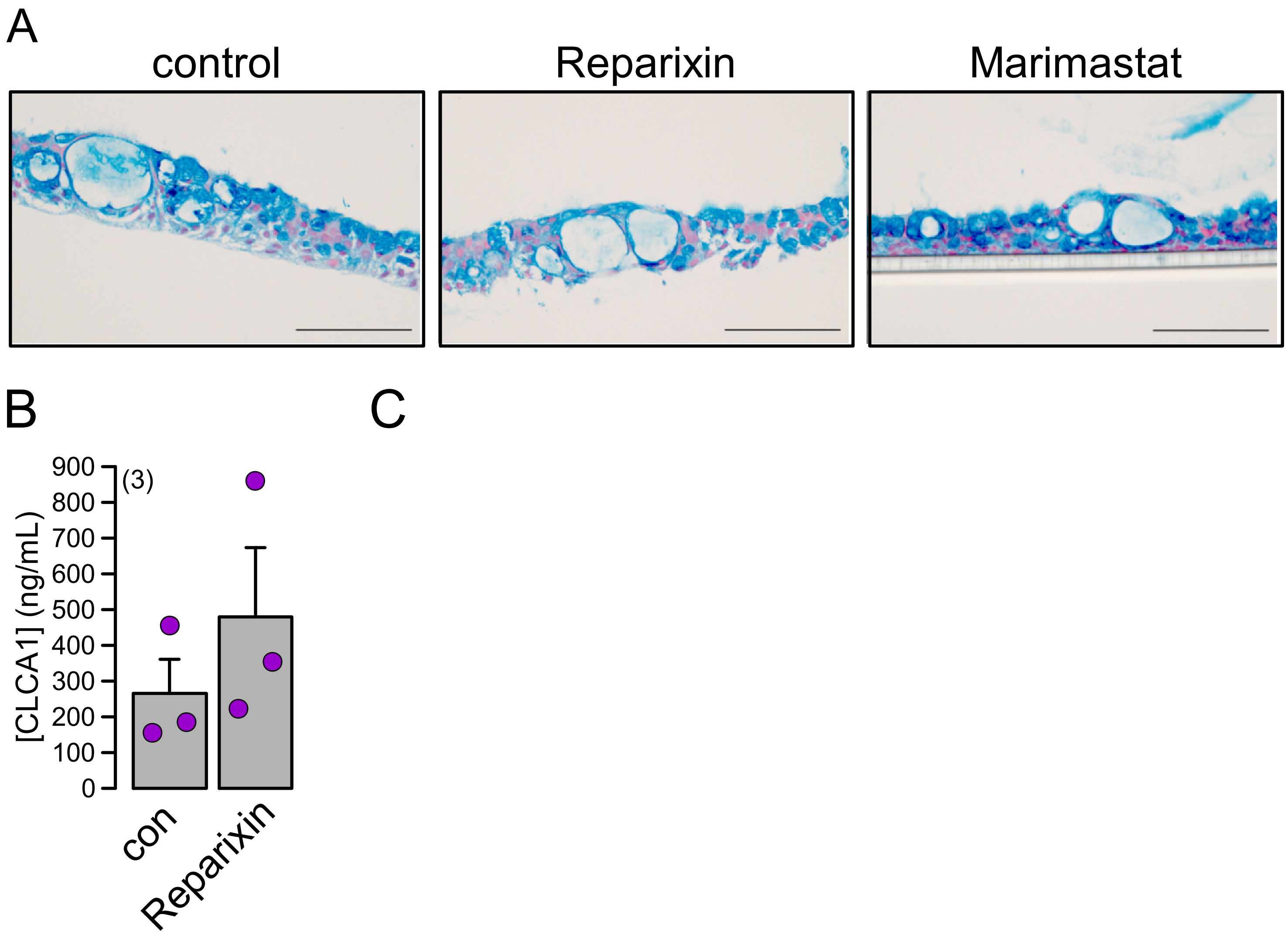

Supplementary Figure 4. *Inhibition of IL-8 signaling or metalloproteinase does not affect goblet cell metaplasia*. A) Inhibition of IL-8 receptors by reparixin (500 nM/30 days) or metalloproteinases by marimastat (150 nM/30 days) does not change the mucosecretory phenotype and hyperplasia of BCi-NS1 epithelia. Bars = 100 µm. B) CLCA1 cytokine release from BCi-NS1 epithelia is not inhibited by blocking IL-8 receptors with reparixin (500 nM/72 hrs).
