# Supplementary material for: TMEM16A/F support exocytosis but do not inhibit Notch-mediated goblet cell metaplasia of BCi-NS1.1 human airway epithelium
Source: Front Physiol. 2023 May 9;14:1157704. doi: 10.3389/fphys.2023.1157704 (PMC10206426; doi:10.3389/fphys.2023.1157704)
Supplement: Supplementary file 4 [file DataSheet6.PDF]

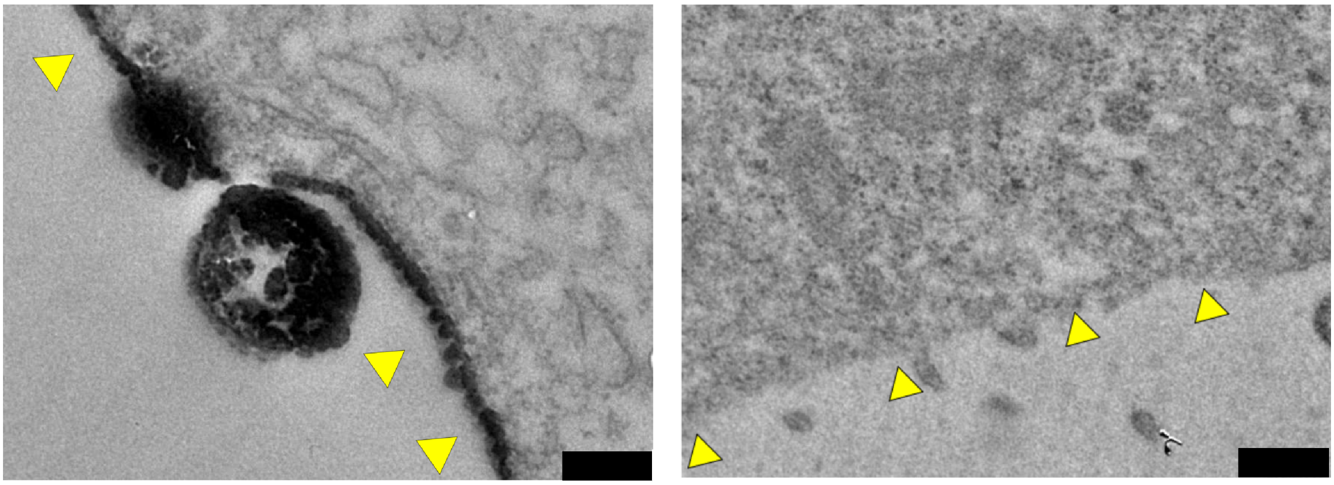

Supplementary Figure 6. *Expression of TMEM16A in COS-7 cells.* A small tetracysteine motif (CCXXCC) was genetically inserted into the target protein TMEM16A, which binds the biarsenical dyes ReAsH. For electron microscopic detection of TMEM16A fused to CCXXCC, photoconversion was performed by exposing the cells at 4°C to a 585-nm light source. TMEM16A is visible as dark precipitation and is expressed in the plasma membrane (yellow arrows) and released extracellular vesicles (exosomes/ectosomes). Bars indicate 200 nm.
