# Supplementary material for: TMEM16A/F support exocytosis but do not inhibit Notch-mediated goblet cell metaplasia of BCi-NS1.1 human airway epithelium
Source: Front Physiol. 2023 May 9;14:1157704. doi: 10.3389/fphys.2023.1157704 (PMC10206426; doi:10.3389/fphys.2023.1157704)
Supplement: Supplementary file 5 [file DataSheet3.PDF]

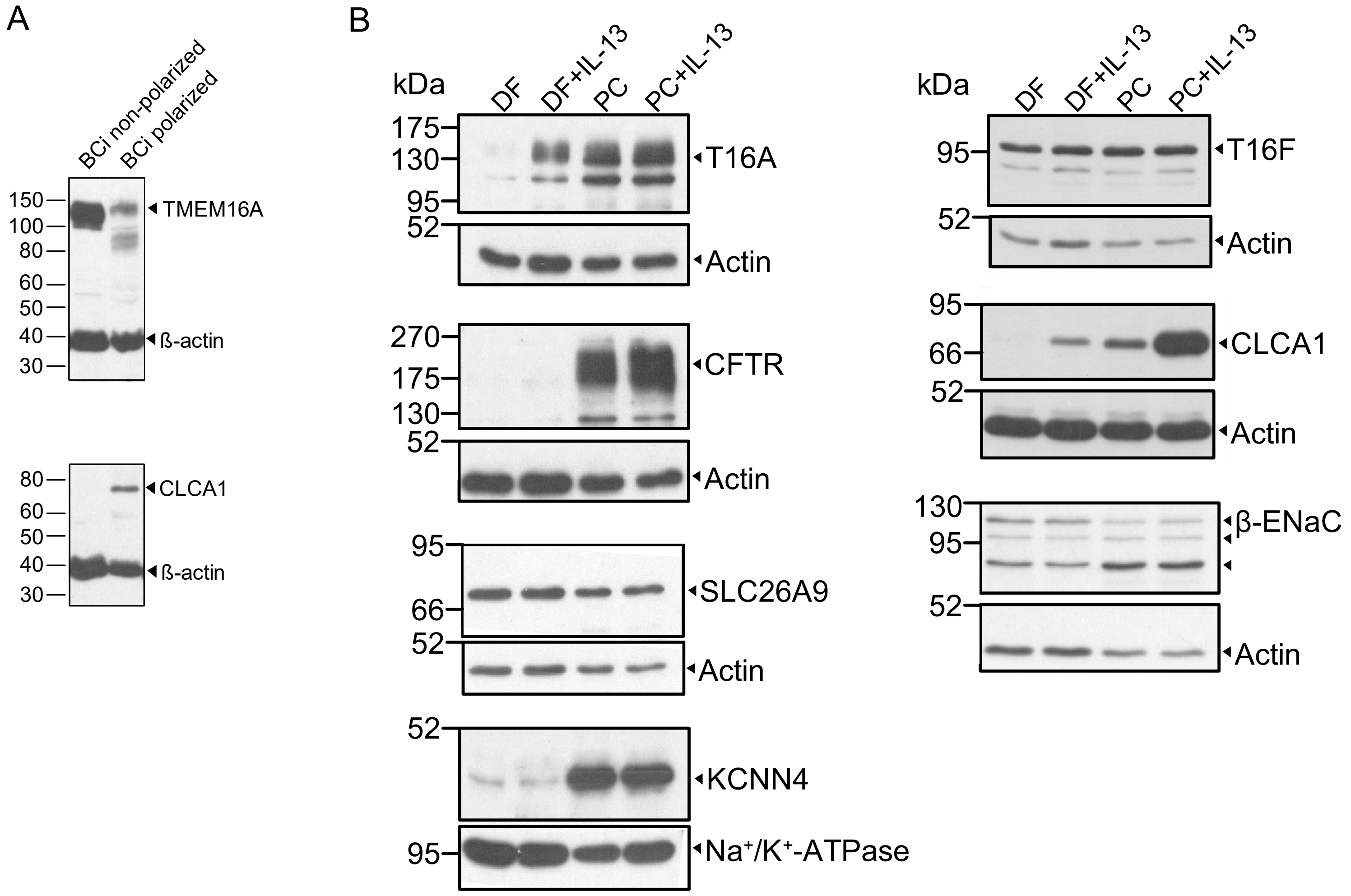

Supplementary Figure 3: *Expression of ion channels in non-polarized and polarized BCi-NS1 cells.* A) Expression of TMEM16A and CLCA1 in BCi-NS1 cells grown under non-polarized and polarized (ALI) conditions in differentiation media (DMEM/Ham's F12 + 2% USG). B) Expression of TMEM16A (T16A), CFTR, slc26A), KCNN4, TMEM16F (T16F), CLCA1, and ß-ENaC grown in ALI in differentiation media (DF; DMEM/Ham's F12 + 2% USG) or PneumaCult TM (PC) media, in the absence or presence of IL-13 (100 ng/ml; 72 hrs). Blots were performed as duplicates.
