# Supplementary material for: TMEM16A/F support exocytosis but do not inhibit Notch-mediated goblet cell metaplasia of BCi-NS1.1 human airway epithelium
Source: Front Physiol. 2023 May 9;14:1157704. doi: 10.3389/fphys.2023.1157704 (PMC10206426; doi:10.3389/fphys.2023.1157704)
Supplement: Supplementary file 6 [file DataSheet1.PDF]

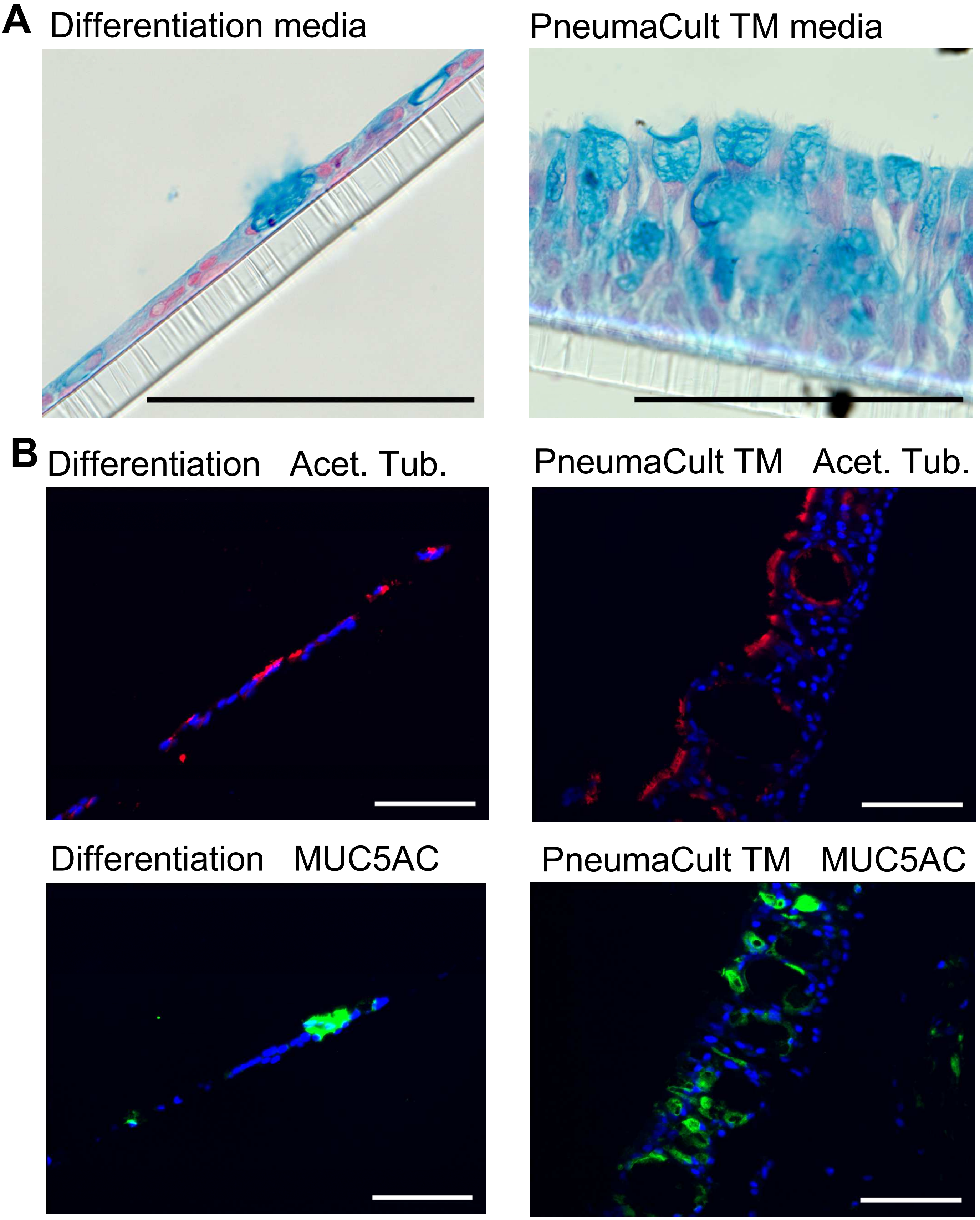

Supplementary Figure 1: *Culture media dependent differentiation of BCi-NS1 human airway epithelial cells.* A) ALI cultures of BCi-NS1 cells grown in differentiation media (DMEM/Ham's F12 + 2% USG) or PneumaCult TM media. B) Immunocytochemistry of acetylated tubulin and MUC5AC of BCi-NS1 cells grown in differentiation media or PneumaCult TM media.
