# Supplementary material for: TMEM16A/F support exocytosis but do not inhibit Notch-mediated goblet cell metaplasia of BCi-NS1.1 human airway epithelium
Source: Front Physiol. 2023 May 9;14:1157704. doi: 10.3389/fphys.2023.1157704 (PMC10206426; doi:10.3389/fphys.2023.1157704)
Supplement: Supplementary file 7 [file DataSheet5.PDF]

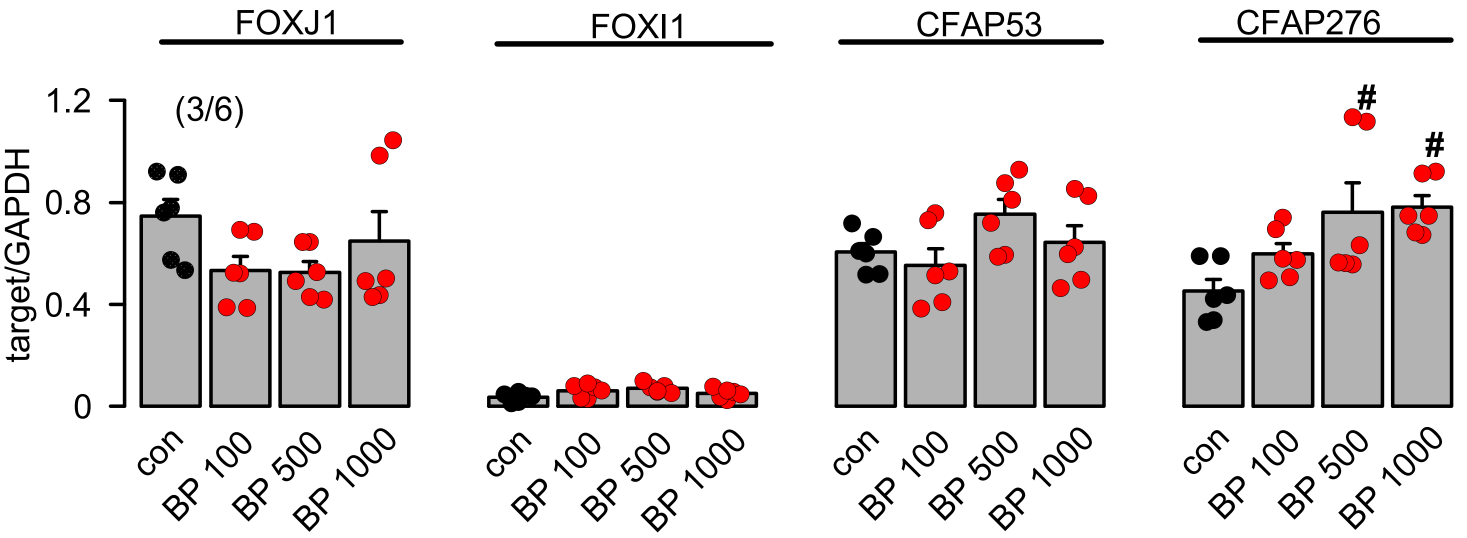

Supplementary Figure 5. *Inhibition of STAT-3 by BP-1-102 does not inhibit differentiation towards ciliated cells and ionocytes.* Semiquantitative RT-PCR analysis of the effect of the STAT-3 inhibitor BP-1-102 (100, 500, 1000 nM for 72 hrs) on expression of the ciliated cell and ionocyte markers FoxJ1, FoxI1, CFAP53, and CFAP276. Mean ± SEM (number of cultures/reactions).
